# Psychosocial factors and coping strategies associated with alcohol use during the COVID-19 pandemic

**DOI:** 10.1192/j.eurpsy.2023.486

**Published:** 2023-07-19

**Authors:** A. Papadopoulou, K. Skyftou, E. Efstathiou, K. Gkikas, E. Kaloudi, P. Bali, K. Papazachos, D. Tsaklakidou, V. Efstathiou

**Affiliations:** ^1^Second Department of Psychiatry, National and Kapodistrian University of Athens, “Attikon” University General Hospital; ^2^Psychology Department, National and Kapodistrian University of Athens; ^3^Marketing and Communication Department, Athens University of Economics and Business, Athens, Greece

## Abstract

**Introduction:**

The investigation of alcohol use and its correlates during the ongoing COVID-19 pandemic is of utmost importance.

**Objectives:**

This study aimed to examine alcohol use during COVID-19 pandemic, while nationwide lockdowns were in effect in Greece, and its relationship with demographic, clinical, and psychosocial factors.

**Methods:**

The study included 378 individuals (225 women) with a mean age of 30.22 years who completed an online questionnaire during the third wave of the pandemic while restriction measures were in effect (March to April 2021). Participants completed Alcohol Use Disorders Identification Test (AUDIT), Fear of COVID-19 Scale (FCV-19S), Depression, Anxiety and Stress Scale (DASS-21), Coping Orientation to Problems Experienced Inventory (Brief-COPE) and the Multidimensional Scale of Perceived Social Support (MSPSS).

**Results:**

According to the results, alcohol use was negatively associated with COVID-19 fear (p = 0.011), and positively associated with anxiety (p = 0.024), depression (p<0.001) and avoidance-focused coping strategies (p = 0.003). Furthermore, perceived social support emerged as a significant protective factor against alcohol use. Men presented higher alcohol use levels compared to women (p = 0.002). Additionally, individuals identified as problematic users, based on AUDIT scores, displayed increased levels of anxiety (p = 0.028) and depression (p = 0.017) and used avoidance-focused coping strategies to a greater extent (p<0.001). Of note, higher alcohol use was observed in participants who lived alone (p<0.001) and in those whose work status had changed during the pandemic (p = 0.004).

**Conclusions:**

Our findings highlight the importance of identifying individuals with problematic alcohol use, as well as recognizing crucial psychosocial factors related to alcohol use especially during the pandemic.

**Disclosure of Interest:**

None Declared

